# Comparison of Short, Intermediate, and Long Cephalomedullary Nail Length Outcomes in Elderly Intertrochanteric Femur Fractures

**DOI:** 10.5435/JAAOSGlobal-D-21-00322

**Published:** 2022-03-02

**Authors:** Tanner N. Womble, Andrew Kirk, Maxwell Boyle, Shea M. Comadoll, Leon Su, Arjun Srinath, Paul Edward Matuszewski, Arun Aneja

**Affiliations:** From the University of Kentucky College of Medicine, Lexington, KY (Dr. Womble, Dr. Kirk, Mr. Boyle, and Mr. Su); the Department of Orthopedic Surgery, University of Minnesota, Minneapolis, MN (Dr. Comadoll); the Department of Orthopaedic Surgery (Dr. Srinath, Dr. Matuszewski, and Dr. Aneja); and the Department of Statistics, University of Kentucky, Lexington, KY. (Mr. Su)

## Abstract

Cephalomedullary nail (CMN) length for intertrochanteric femur fractures without subtrochanteric extension has been an ongoing debate. The authors hypothesize that increasing nail length would result in increasing surgical time, greater incidence of acute kidney injury (AKI), postoperative anemia, and blood loss requiring transfusion due to increased intramedullary reaming and pressurization of the canal with nail insertion.

**Methods:**

A retrospective chart review of patients aged 65 years or older who underwent CMN for low-energy intertrochanteric femur fractures from 2010 to 2018 was undertaken. Patient demographic data, comorbidities, case duration, postoperative hospital length of stay (LOS), and laboratory data, including serum creatinine, hemoglobin, and hematocrit, were collected for analysis. The following outcome measures were compared: postoperative pneumonia, cardiac complications, sepsis, reintubation/intensive care unit stay, pulmonary embolism, stroke, postoperative AKI, 30-day hospital readmission, 30-day return to operating room, 30-day mortality, 1-year mortality, postoperative anemia (hemoglobin <7 g/dL), and blood transfusion.

**Results:**

A total of 247 patients were analyzed (short = 48, intermediate = 39, and long = 160). No notable difference was observed in postoperative pneumonia, cardiac complications, sepsis, reintubation/intensive care unit stay, pulmonary embolism, stroke, mean total hospital LOS, mean postoperative hospital LOS, rate of postoperative AKI, 30-day readmission, 30-day return to operating room, 30-day mortality, or 1-year mortality. Patients receiving long nails had significantly higher rates of postoperative anemia (*P* = 0.0491), blood transfusion (*P* = 0.0126), and mean procedure length (*P* = 0.0044) compared with the two other groups.

**Discussion:**

Patients receiving long nails had markedly higher rates of postoperative anemia and blood loss requiring blood transfusion with markedly longer mean procedure length than patients receiving short and intermediate CMNs. Long nails did not result in an increase in other complications evaluated.

Intertrochanteric femur fractures have been identified as a major health concern in developed nations.^1^ Most of these fractures occur in the olderly patients, leading to markedly increased morbidity and mortality and consequential decrease in quality of life.^2,3,4,5^ Increasing rates of osteoporosis in the setting of an aging population further add to the health burden because the number of low-energy intertrochanteric femur fractures is expected to rise.^1,6^

Cephalomedullary nailing (CMN) is the most common method for treating displaced intertrochanteric femur fractures.^3,7-9^ The choice of nail length has remained a controversial topic with multiple studies comparing short versus long cephalomedullary nails.^3,10,11,12,13,14,15,16^ Advantages proposed in favor of short CMNs include ease of use, decreased surgical time and blood loss, decreased implant cost, and targeted locking bolts through the insertion jig with the disadvantage of spanning a shorter segment of the femur in patients with a pathologic osteoporotic bone and history of falls.^13^ This has led to the theoretical proposition that long-length CMNs hold an advantage through the protection of the entire femoral shaft but hold the disadvantages of increased operation times, increased need for reaming, increased cost, need for free-hand distal locking, and mismatch of nail bow to femur risking distal anterior cortical penetration with certain older generation implants.^13^ Despite several studies comparing these, a firm conclusion regarding the morbidity of increased cephalomedullary reaming and longer canal instrumentation has yet to be reached regarding nail choice. In addition, to the authors' knowledge, there is limited mention of intermediate-length nails in these comparison studies.

The objective of this investigation was to retrospectively compare clinical outcomes in olderly patients with low-energy intertrochanteric femur fractures without subtrochanteric involvement after treatment with a short, intermediate, or long cephalomedullary nail. The authors hypothesized that an increasing nail length would result in increasing surgical time, greater incidence of acute kidney injury (AKI), postoperative anemia, and blood loss requiring transfusion due to increased intramedullary reaming and pressurization of the canal with nail insertion. To the best of our knowledge, there are only a limited number of studies that include intermediate-length nails in the comparison of cephalomedullary nails.^17,18^

## Methods

As part of this Institutional Review Board-approved retrospective study, we identified patients who underwent CMN for intertrochanteric femur fractures from 2010 to 2018 at a single level I trauma center. Patients were included if they were 65 years or older with an OTA 31-A (1-3) fracture pattern and more than 1-year follow-up.^19^ Patients were excluded if they were polytrauma patients or had their hip fractures classified as subtrochanteric or secondary to a pathologic lesion. Patient demographic data, American Society of Anesthesiologists grade, case duration, postoperative hospital length of stay (LOS), along with comorbidities, including diabetes, hypertension, congestive heart failure, chronic kidney disease, chronic obstructive pulmonary disease, tobacco use, and obesity (body mass index >30), were collected. In addition, preoperative and postoperative day 1 laboratory data, including serum creatinine, hemoglobin, and hematocrit, were collected. Preoperative and postoperative patient radiographs were reviewed for nail length, and fractures were classified using the AO/OTA fracture classification guidelines. Patients were then compared in three groups based on nail length (short, intermediate, and long) for the analysis of primary outcome measures, which included procedure length defined as an incision to closure, incidence of hospital readmission within 30 days, return to operating room (OR) within 30 days, 30-day mortality, 1-year mortality, postoperative anemia defined as hemoglobin <10 g/dL, and blood transfusion which at our institution is administered at hemoglobin <7 g/dL. In patients requiring blood transfusion, the number of units transfused was based on managing physician preference with a goal of maintaining hemoglobin >7 g/dL. Groups were additionally analyzed for differences in the following postoperative complications: stroke, cardiac complication (arrhythmia, arrest, or infarct), pneumonia, requirement of reintubation or intensive care unit stay, pulmonary embolism, any other pulmonary complication (hypoxia, acute hypoxemic respiratory failure, or requirement of supplemental oxygen), sepsis, and AKI defined by the Kidney Disease Improving Global Outcomes criteria.^20^

All surgeries were done by trauma fellowship-trained attending physicians. In all cases, residents or fellows participated as the first assist. All patients received intramedullary reaming before nail insertion because this is the standard of practice at our institution. The entire length of the canal was reamed for long nails, whereas for intermediate and short nails, only the necessary length of the canal was reamed. Only DePuy Synthes Titanium Trochanteric Fixation Nails were used. Nail length classification was defined by the following: 170 mm as short, 235 mm as intermediate, and 300 to 460 mm as long. The decision of nail length was made on the basis of surgeon preference, with clinical indication taken into account. All patients were made weight-bearing as tolerated postoperatively. The standard anterior-posterior and lateral radiograph images were obtained postoperatively and at 6 weeks, 12 weeks, 6 months, and 1 year.

Pearson chi-square and Fisher exact test were used to analyze the association between categorical variables and nail length. One-way analysis of variance was used to analyze the association between continuous variables and nail length. Post hoc multiple comparisons were done for continuous variables found to have a notable association with nail length. Multiple comparisons were done using the protected least notable difference method. The level of statistical significance used was 0.05, with a Bonferroni correction applied for multiple comparisons. All analyses were completed using SAS 9.4 (SAS Institute).

## Results

### Patient Demographics

**Figure 1:**
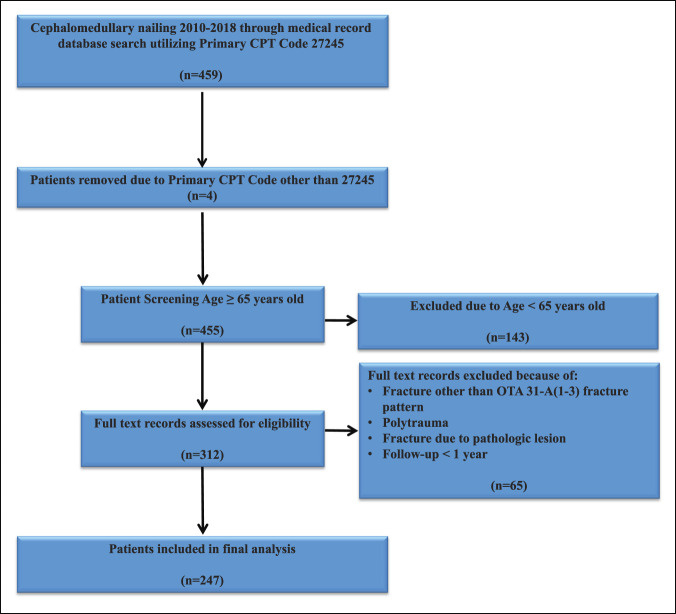
Inclusion and exclusion criteria. CPT = current procedural terminology

A total of 247 patients met inclusion criteria (short = 48, intermediate = 39, and long = 160) (Figure 1). The average age of patients (short = 80.08, intermediate = 79.23, and long = 80.97, *P* = 0.4241) was not significantly different among nail groups. In addition, the rates of congestive heart failure (*P* = 0.355), chronic kidney disease (*P* = 0.327), obesity (*P* = 0.153), and tobacco use (*P* = 0.246) did not differ among the study population. Conversely, patients with the following comorbidities, such as diabetes (short = 20.8% versus intermediate = 46.2% versus long = 26.9%, *P* = 0.023), chronic obstructive pulmonary disease (short = 22.9% versus intermediate = 23.1% versus long = 9.4%, *P* = 0.014), and hypertension (short = 68.8% versus intermediate = 87.2% versus long = 66.9%, *P* = 0.036), were more likely to receive an intermediate-length nail. When comparing nail length and fracture classification, a short-length nail was significantly more likely to be used in OTA 31A1 fracture patterns, whereas long-length nails were significantly more likely to be used in OTA 31A2 and 31A3 fracture patterns (Tables 1–3).

**Table 1:** Patient Demographics: Sex, Age, and OTA Fracture Classification Extrapolated by Nail Length

Demographics	Short (n = 48)	Intermediate (n = 39)	Long (n = 160)	*P* Value
Sex, n (%)				
Male	13 (27.1)	11 (28.2)	41 (25.6)	0.9391
Female	35 (72.9)	28 (71.8)	119 (74.4)	
Age^a^ (yrs)	80.08 (8.81)	79.13 (7.36)	81.03 (7.67)	0.4241
OTA fracture classification				
31A1	33 (68.8)	23 (59.0)	73 (45.6)	**0.0086**
31A2	12 (25.0)	12 (30.8)	47 (29.4)	
31A3	3 (6.3)	4 (10.3)	40 (25.0)	

a Continuous variables are formatted as follows: mean (SD). Bold entries are statistically significant at p≤0.05.

**Table 2:** Patient Demographics: Comorbidities Extrapolated by Nail Length

Comorbidities	Short (n = 48)	Intermediate (n = 39)	Long (n = 160)	*P* Value
Diabetes, n (%)	10 (20.8)	18 (46.2)	43 (26.9)	**0.0234**
Hypertension, n (%)	33 (68.8)	34 (87.2)	107 (66.9)	**0.0357**
Congestive heart failure, n (%)	7 (14.6)	10 (25.6)	27 (16.9)	0.3551
COPD, n (%)	11 (22.9)	9 (23.1)	15 (9.4)	**0.0136**
Obesity, n (%)	3 (6.3)	2 (5.1)	3 (1.9)	0.1532
Tobacco use, n (%)	10 (20.8)	7 (17.9)	19 (11.9)	0.2462
CKD, n (%)	18 (37.5)	10 (27.0)	64 (40.3)	0.3269

CKD = chronic kidney disease, COPD = chronic obstructive pulmonary disease. Bold entries are statistically significant at p≤0.05.

**Table 3:** Post Hoc Fracture Classification Comparison for Different Nail Lengths

Nail length	A1 Versus A2	A1 Versus A3	A2 Versus A3
Short	0.1595	**0.0053**	0.0931
Intermediate	0.8687	0.1291	0.1925
Long	0.1844	**0.0004**	**0.0223**

Bold entries are statistically significant at p≤0.05.

### Nail Length Outcomes

Our analysis indicated that long nails had significantly higher rates of postoperative anemia (short = 14.6% versus intermediate = 2.6% versus long = 16.9%, *P* = 0.0491) and blood loss requiring transfusion (short = 33.3% versus intermediate = 25.6% versus long = 48.8%, *P* = 0.013) compared with both short and intermediate nails. Procedure length was also shown to be significantly greater in patients receiving long-length nails (short = 66.38 mins versus intermediate = 67.13 mins versus long = 79.38 mins, *P* = 0.004). Post hoc analysis was done for mean procedure length, showing the differences between long nails versus intermediate nails (*P* = 0.0163) and long nails versus short nails (*P* = 0.0062) were significant, whereas the difference between short versus intermediate nails remained insignificant (*P* = 0.903). No significant difference was observed in preoperative hemoglobin <7 (*P* = 0.7875), preoperative hemoglobin <10 (*P* = 0.1324), change in hematocrit post-pre (*P* = 0.6655), change in hemoglobin post-pre (*P* = 0.9564), estimated blood loss (EBL) (*P* = 0.956), mean total hospital LOS (short = 13.79 days versus intermediate = 8.85 days versus long = 8.73 days, *P* = 0.1708), or mean postoperative hospital LOS (short = 12.73 days versus intermediate = 7.28 days versus long = 7.36 days, *P* = 0.129) between different CMN lengths. Similarly, nail length did not influence the rate of AKI (*P* = 0.272), readmission within 30 days (*P* = 1.00), or return to OR within 30 days (*P* = 1.00). Furthermore, there was no significant difference in the following postoperative complications: stroke (*P* = 1.00), cardiac complication (arrhythmia, arrest, or infarct) (*P* = 0.314), postoperative pneumonia (*P* = 0.177), requirement of reintubation or intensive care unit stay (*P* = 0.941), pulmonary embolism (*P* = 0.788), and sepsis (*P* = 0.242) (Tables 4–7).

**Table 4:** Postoperative Outcome Comparison of Individual Nail Lengths

Outcome	Short (n = 48)	Intermediate (n = 39)	Long (n = 160)	*P* Value
Procedure length^a^ (min)	66.38 (22.33)	67.13 (27.25)	79.38 (30.06)	**0.0044**
Postoperative length of stay^a^	12.73 (34.90)	7.28 (4.97)	7.36 (7.14)	0.1294
Total length of stay^a^	13.79 (34.76)	8.85 (6.39)	8.73 (7.55)	0.1708
Return to inpatient within 30 d	2 (4.2%)	2 (5.1%)	8 (5.0%)	1.0000
Return to operating room within 30 d	0 (0.0%)	0 (0.0%)	2 (1.3%)	1.0000
Death within 30 d	4 (8.3%)	4 (10.3%)	14 (8.8%)	0.9461
Death within 1 yr^b^	10 (20.8%)	5 (13.2%)	19 (11.9%)	0.2893

a Continuous variables are formatted as follows: mean (SD).

b Data from 1 patient were missing for death within 1 year. Bold entries are statistically significant at p≤0.05.

**Table 5:** Postoperative Complications Comparison of Individual Nail Lengths

Postoperative Complications	Short (n = 48)	Intermediate (n = 39)	Long (n = 160)	*P* Value
Postoperative anemia	7 (14.6%)	1 (2.6%)	27 (16.9%)	**0.0491**
Postoperative AKI^a^	8 (17.8%)	2 (5.7%)	21 (14.3%)	0.2719
Postoperative pneumonia	0 (0.0%)	1 (2.6%)	10 (6.3%)	0.1769
Pulmonary embolism	0 (0.0%)	0 (0.0%)	4 (2.5%)	0.7875
Cardiac complication	2 (4.2%)	2 (5.1%)	3 (1.9%)	0.3142
Stroke	0 (0.0%)	0 (0.0%)	1 (0.6%)	1.0000
Sepsis	0 (0.0%)	2 (5.1%)	4 (2.5%)	0.2424
Blood transfusion	16 (33.3%)	10 (25.6%)	78 (48.8%)	**0.0126**
Estimated blood loss (mL)^b^	150.38 (90.08)	148.75 (114.26)	153.62 (90.74)	0.9564

AKI = acute kidney injury

a Data from 20 patients were missing for postoperative AKI. ^b^Continuous variables are formatted as follows: mean (SD). Bold entries are statistically significant at p≤0.05.

**Table 6:** Additional Analysis of Preoperative and Postoperative Hgb and Hct

Hgb and Hct	Short (n = 48)	Intermediate (n = 39)	Long (n = 160)	*P* Value
Preoperative Hgb <7	0 (0.0%)	0 (0.0%)	4 (2.5%)	0.1324
Preoperative Hgb <10	16 (33.3%)	8 (20.5%)	60 (37.5%)	0.3269
Change in Hct post-pre^a^	−6.37 (3.88)	−5.31 (4.06)	−5.81 (5.43)	0.6655
Change in Hgb post-pre^a^	−2.09 (1.35)	−1.78 (1.35)	−1.88 (1.84)	0.9564

Hct = hematocrit, Hgb = hemoglobin

a Continuous variables are formatted as follows: mean (SD).

**Table 7:** Post Hoc Outcome Comparisons for Different Nail Lengths Procedure Lengths

Nail length 1	Nail Length 2	Estimate of 1 to 2	*P*
Long	Intermediate	12.2524	**0.0163**
Long	Short	12.9977	**0.0062**
Intermediate	Short	0.7452	0.9033

Bold entries are statistically significant at p≤0.05.

## Discussion

Extensive literature comparing long and short CMNs exists; however, this is the first study to directly compare intermediate-length CMNs versus short-length and long-length CMNs. Historically, long nails have shown to be more prevalent in the cephalomedullary fixation of intertrochanteric femur fractures, and the comparatively high rate of long nail use in our study was consistent with this.^3,10,11,12,13,15,16,21-23^ Two patients receiving long nails required return to the OR within 30 days, one because of postoperative bleeding and the other because of a contralateral hip fracture. Similar to previous findings of Krigbaum et al, the results of this retrospective study emphasize that there was no notable difference in the primary outcome measures of readmission within 30 days or return to OR within 30 days between nail groups.^10,12,24,25^ AKI, which has not been examined in previous nail length comparison studies, was selected as an outcome measure due to the prevalence of AKI after surgery for hip fracture repair.^26-28^ The authors also chose to examine the rates of AKI due to the concern of the effect of systemic emboli from pressurization of the intramedullary canal during reaming and nail insertion. The increased duration and length of reaming along with the increased length of nail insertion with long CMN could translate to a clinically meaningful difference.^29,30^ However, our results found that there was no notable difference in AKI rates between nail lengths.

A multitude of previous studies have reported higher EBL in long nails.^3,10,11,18,24,25,31^ The authors of this study chose postoperative anemia as a primary end point over EBL because of its general inaccuracy and inaccuracy in predicting need for transfusion.^32-34^ Furthermore, postoperative anemia is associated with early postoperative functional status impairment, increased urinary and respiratory tract infections, increased hospital LOS, and increased mortality in both the 30-day and 12-month postoperative periods.^35^ The definition of postoperative anemia varies between institutions ranging from stringent (hemoglobin level less than 10g/dL) to liberal (hemoglobin level less than 13g/dL) according to a systematic review by Spahn.^35^ Hemoglobin less than 10g/dL was used for this study because that is our institutional practice. EBL was not markedly different; however, we found a statistically significant higher rate of postoperative anemia in patients receiving long-length nails. Blood loss requiring transfusion between nail lengths was found to be markedly higher in patients receiving long nails, which aligns with our postoperative anemia results. This is in line with Boone et al and Zhang et al who found markedly higher rates of transfusion in patients receiving long nails.^3,24^ The rates of blood transfusion in intermediate nail length patients are nearly identical to the findings in a case series examining intermediate-length nails by Enns et al.^17^ However, Thamyongkit et al^18^ comparing intermediate versus long-length nails found no notable difference in the rates of blood transfusion despite markedly higher rates of intraoperative blood loss in long-length nails.

Previous studies comparing short versus long-length nails have shown mixed results for hospital LOS, with some reporting a markedly longer hospital LOS when long nails were used while others found no notable difference.^3,12,13,24^ We did not find notable difference in total LOS or postoperative LOS between the nail lengths. Earlier studies have shown consistently longer surgical time for long nails, which supports the results of our study.^3,10,11,15,18,24,25,31,36,37^ The procedure lengths recorded in our study were from the time of incision until closure. Every patient received two distal interlocking screws, which may have been a contributing factor to the difference in surgical time observed. Both the short and intermediate nails used allowed for the use of a guide for distal interlocking screw placement, whereas the long-length nails required freehand placement. This has been shown by Kleweno et al^15^ to be a primary factor in the increased surgical time seen with long nails. Post hoc analysis confirmed that long nails had markedly longer procedure length than both intermediate and short nails, whereas intermediate nail procedure length was not markedly different from short nails. In addition, there were no occurrences of periimplant fracture at the 1-year point, we believe this was due to all patients receiving distal interlocking screws because these have been shown to protect against future fractures at or distal to the stem.^38^

Mortality from surgical treatment of intertrochanteric fractures typically results from cardiopulmonary complications, thromboembolism, and/or sepsis. This led to our selection of the adverse perioperative and postoperative outcomes analyzed in this study.^39^ Despite notable differences in demographics existing in our study population, our results of no notable difference in adverse outcomes are in concordance with existing evidence, which has shown no notable difference in mortality, pulmonary complications, or other notable morbidities.^10,12,18,24,25,37^ Overall, 22 patients died within 30 days (8.9%) and 34 patients within 1 year (13.8%) in our study population, which is consistent with the estimated rates of 14% to 36%, the 1-year mortality rate in existing evidence.^40^ The use of intermediate nails was not associated with notable difference in mortality or postoperative complications despite patients who received intermediate-length nails having a statistically significantly higher rate of comorbidities.

This study is limited by the associated biases of its retrospective design. We did not run a priori power analysis because we felt that we had an adequate sample size to detect a difference, but the quantity of both intermediate and short-length nails that met the inclusion and exclusion criteria cannot be ignored as a limitation. A post hoc power analysis was conducted at the 0.05 significance level and a power of 80%, which showed that our study was not adequately powered to detect differences present in our data for the outcomes of pulmonary embolism, postoperative pneumonia, or postoperative AKI. The power analysis indicated to detect a difference, a total population of 1061 patients for pulmonary embolism, 621 patients for postoperative pneumonia, and 848 patients for AKI were necessary. In addition, data on 8% (20/247) of patients were missing regarding AKI, further highlighting the lack of power to detect differences in the rates of postoperative AKI between the three nail lengths. Nonetheless, this study reports all patients who had at least 1-year follow-up, which decreased the sample size but allowed us to present more meaningful data. Although the authors chose to report mortality data, selection bias due to loss to follow-up exists and must be taken into account. In addition, owing to the retrospective study design, we did not have access to uniform postoperative functional status scores and thus were unable to report them. Similarly, owing to the retrospective design, there was no uniformity in the timing of postoperative laboratory analyses, with some occurring in the PACU postprocedure while others occurred on postoperative day 1. The lack of inclusion of cost analysis is another limitation of this study; however, the pricing for a long cephalomedullary nail usually exceeds that of an intermediate or short cephalomedullary nail. Surgeon preference toward the use of a specific nail length for a specific fracture pattern is a large hidden bias in our retrospective data. Furthermore, the inclusion of OTA 31-A3 fracture patterns could have been a circumstance that encourages some surgeons to avoid the use of a short nail. We believe this effect to be minimal; however, because Shannon et al^25^ found no notable difference, when comparing a long nail cohort with a short nail cohort, in periimplant fracture or lag screw cutout, with the short nail cohort tolerating up to 3 cm of subtrochanteric fracture line extension. Furthermore, we did not collect or examine surgeon experience nor account for resident and fellow participation when evaluating the outcome data. We expect a uniform distribution of surgeon experience level and resident and fellow participation level; however, this was still a limitation of this study. This study adds evidence to the existing literature comparing short and long CMNs while spotlighting the less-examined intermediate-length of device. Given the retrospective design of most published studies, the small sample size of earlier prospective studies, and the relative paucity of data on functional status, additional study of the outcomes of short, intermediate, and long nails is required.

## Conclusion

Patients receiving long CMNs had markedly higher rates of postoperative anemia and blood loss requiring blood transfusion with markedly longer mean procedure length than patients receiving short and intermediate CMNs for low-energy intertrochanteric femur fractures. Our study adds to existing evidence that the use of both short and long implants is viable options while bringing to evidence that intermediate nails can be considered as a viable alternative because their use did not perform markedly worse in any measured outcome. Ultimately, treatment should be tailored to the patient and the surgeon's ability to perform the procedure in a safe manner. Additional studies, both prospective and/or with greater statistical power, are needed to further evaluate the significance of nail length on postoperative outcomes.

## References

[1] Brauer CA Coca-Perraillon M Cutler DM Rosen AB: Incidence and mortality of hip fractures in the United States. JAMA 2009;302:1573-1579. 19826027 10.1001/jama.2009.1462 PMC4410861

[2] Bliuc D Nguyen ND Milch VE Nguyen TV Eisman JA Center JR: Mortality risk associated with low-trauma osteoporotic fracture and subsequent fracture in men and women. JAMA 2009;301:513-521. 19190316 10.1001/jama.2009.50

[3] Boone C Carlberg KN Koueiter DM : Short versus long intramedullary nails for treatment of intertrochanteric femur fractures (OTA 31-A1 and A2). J Orthop Trauma 2014;28:e96-e100. 24751609 10.1097/BOT.0b013e3182a7131c

[4] Cummings SR Melton LJ: Epidemiology and outcomes of osteoporotic fractures. Lancet 2002;359:1761-1767. 12049882 10.1016/S0140-6736(02)08657-9

[5] Elmi A Rohani AR Tabrizi A Esmaili SM: Comparison of outcome of femoral shaft fracture fixation with intramedullary nail in elderly patient and patients younger than 60 years old. Arch Bone Jt Surg 2014;2:103-105. 25207327 PMC4151446

[6] Marks R: Hip fracture epidemiological trends, outcomes, and risk factors, 1970-2009. IJGM 2010;3:1-17. PMC2866546 20463818

[7] Nie B Wu D Yang Z Liu Q: Comparison of intramedullary fixation and arthroplasty for the treatment of intertrochanteric hip fractures in the elderly. Medicine (Baltimore) 2017;96:e7446. 28682912 10.1097/MD.0000000000007446 PMC5502185

[8] Radcliff TA Regan E Cowper Ripley DC Hutt E: Increased use of intramedullary nails for intertrochanteric proximal femoral fractures in veterans affairs hospitals: A comparative effectiveness study. J Bone Joint Surg Am 2012;94:833-840. 22552673 10.2106/JBJS.I.01403

[9] Kokoroghiannis C Aktselis I Deligeorgis A Fragkomichalos E Papadimas D Pappadas I: Evolving concepts of stability and intramedullary fixation of intertrochanteric fractures—A review. Injury 2012;43:686-693. 21752370 10.1016/j.injury.2011.05.031

[10] Hou Z Bowen TR Irgit KS : Treatment of pertrochanteric fractures (OTA 31-A1 and A2): Long versus short cephalomedullary nailing. J Orthop Trauma 2013;27:7. 10.1097/BOT.0b013e31826fc11f 22955331

[11] Frisch NB Nahm NJ Khalil JG Les CM Guthrie ST Charters MA: Short versus long cephalomedullary nails for pertrochanteric hip fracture. Orthopedics 2017;40:83-88. 27874910 10.3928/01477447-20161116-01

[12] Krigbaum H Takemoto S Kim HT Kuo AC. Costs and complications of short versus long cephalomedullary nailing of OTA 31-A2 proximal femur fractures in U.S. Veterans. J Orthop Trauma 2016;30:125-129. 26894639 10.1097/BOT.0000000000000521

[13] Baldwin PC Lavender RC Sanders R Koval KJ. Controversies in intramedullary fixation for intertrochanteric hip fractures. J Orthop Trauma 2016;30:635-641. 27437614 10.1097/BOT.0000000000000652

[14] Niu E Yang A Harris AHS Bishop J: Which fixation device is preferred for surgical treatment of intertrochanteric hip fractures in the United States? A survey of orthopaedic surgeons. Clin Orthop Relat Res 2015;473:3647-3655. 26208608 10.1007/s11999-015-4469-5 PMC4586189

[15] Kleweno C Morgan J Redshaw J : Short versus long cephalomedullary nails for the treatment of intertrochanteric hip fractures in patients older than 65 years. J Orthop Trauma 2014;28:391-397. 24231580 10.1097/BOT.0000000000000036

[16] Kanakaris NK Tosounidis TH Giannoudis PV. Nailing intertrochanteric hip fractures: Short versus long; locked versus nonlocked. J Orthop Trauma 2015;29(suppl 4):S10-S16. 10.1097/BOT.0000000000000286 25756821

[17] Enns PA Nyberg SM Berg GM : Clinical outcomes of intermediate-length cephalomedullary nails for intertrochanteric femur fracture repair in older adults. Kans J Med 2020;13:106-111. 32499863 PMC7266504

[18] Thamyongkit S MacKenzie JS Sirisreetreerux N Shafiq B Hasenboehler EA: Outcomes after unstable pertrochanteric femur fracture: Intermediate versus long cephalomedullary nails. Eur J Trauma Emerg Surg 2020;46:963-968. 30143808 10.1007/s00068-018-1002-z

[19] Marsh JL Slongo TF Agel J : Fracture and dislocation classification compendium—2007: Orthopaedic Trauma Association classification, database and outcomes committee. J Orthop Trauma 2007;21(10 suppl):S1-S133. 18277234 10.1097/00005131-200711101-00001

[20] Khwaja A: KDIGO clinical practice guidelines for acute kidney injury. Nephron 2012;120:c179-c184. 22890468 10.1159/000339789

[21] Anglen JO Weinstein JN: American Board of Orthopaedic Surgery Research Committee. Nail or plate fixation of intertrochanteric hip fractures: Changing pattern of practice. A review of the American Board of Orthopaedic Surgery Database. J Bone Joint Surg Am 2008;90:700-707. 18381305 10.2106/JBJS.G.00517

[22] Bjørgul K Reikerås O: Outcome after treatment of complications of Gamma nailing: A prospective study of 554 trochanteric fractures. Acta Orthop 2007;78:231-235. 17464612 10.1080/17453670710013735

[23] Sehat K Baker RP Pattison G Price R Harries WJ Chesser TJS: The use of the long gamma nail in proximal femoral fractures. Injury 2005;36:1350-1354. 16054630 10.1016/j.injury.2005.03.003

[24] Zhang Y Zhang S Wang S : Long and short intramedullary nails for fixation of intertrochanteric femur fractures (OTA 31-A1, A2 and A3): A systematic review and meta-analysis. Orthop Traumatol Surg Res 2017;103:685-690. 28546048 10.1016/j.otsr.2017.04.003

[25] Shannon S Yuan B Cross W : Short versus long cephalomedullary nails for pertrochanteric hip fractures: A randomized prospective study. J Orthop Trauma 2019;33:480-486. 31232891 10.1097/BOT.0000000000001553

[26] Hong SE Kim TY Yoo JH : Acute kidney injury can predict in-hospital and long-term mortality in elderly patients undergoing hip fracture surgery. PLoS One 2017;12:e0176259. 28426743 10.1371/journal.pone.0176259 PMC5398695

[27] Porter CJ Moppett IK Juurlink I Nightingale J Moran CG Devonald MAJ: Acute and chronic kidney disease in elderly patients with hip fracture: Prevalence, risk factors and outcome with development and validation of a risk prediction model for acute kidney injury. BMC Nephrol 2017;18:20. 28088181 10.1186/s12882-017-0437-5 PMC5237525

[28] Ulucay C Eren Z Kaspar EC : Risk factors for acute kidney injury after hip fracture surgery in the elderly individuals. Geriatr Orthop Surg Rehabil 2012;3:150-156. 23569709 10.1177/2151458512473827 PMC3598517

[29] Giannoudis PV Tzioupis C Pape HC: Fat embolism: The reaming controversy. Injury 2006;37(4 suppl):S50-S58. 16990061 10.1016/j.injury.2006.08.040

[30] Rothberg DL Makarewich CA: Fat embolism and fat embolism syndrome. J Am Acad Orthop Surg 2019;27:e346-e355. 30958807 10.5435/JAAOS-D-17-00571

[31] Sadeghi C Prentice HA Okike KM Paxton EW: Treatment of intertrochanteric femur fractures with long versus short cephalomedullary nails. Perm J 2020;24:19.229. 10.7812/TPP/19.229 PMC7357999 32663128

[32] Guinn NR Broomer BW White W Richardson W Hill SE: Comparison of visually estimated blood loss with direct hemoglobin measurement in multilevel spine surgery. Transfusion 2013;53:2790-2794. 23438094 10.1111/trf.12119

[33] Goodnough LT Panigrahi AK: Estimating blood loss. Anesth Analg 2017;125:13-14. 28628574 10.1213/ANE.0000000000002121

[34] Lopez-Picado A Albinarrate A Barrachina B: Determination of perioperative blood loss: Accuracy or approximation? Anesth Analg 2017;125:280-286. 28368940 10.1213/ANE.0000000000001992

[35] Spahn DR: Anemia and patient blood management in hip and knee surgery: A systematic review of the literature. Anesthesiology 2010;113:482-495. 20613475 10.1097/ALN.0b013e3181e08e97

[36] Vaughn J Cohen E Vopat BG Kane P Abbood E Born C: Complications of short versus long cephalomedullary nail for intertrochanteric femur fractures, minimum 1 year follow-up. Eur J Orthop Surg Traumatol 2015;25:665-670. 25337958 10.1007/s00590-014-1557-2

[37] Okcu G Ozkayin N Okta C Topcu I Aktuglu K: Which implant is better for treating reverse obliquity fractures of the proximal femur: A standard or long nail? Clin Orthop Relat Res 2013;471:2768-2775. 23564362 10.1007/s11999-013-2948-0 PMC3734419

[38] Lindvall E Ghaffar S Martirosian A Husak L: Short versus long intramedullary nails in the treatment of pertrochanteric hip fractures: Incidence of ipsilateral fractures and costs associated with each implant. J Orthop Trauma 2016;30:119-124. 26270458 10.1097/BOT.0000000000000420

[39] Kaplan K Miyamoto R Levine BR Egol KA Zuckerman JD: Surgical management of hip fractures: An evidence-based review of the literature. II: Intertrochanteric fractures. J Am Acad Orthop Surg 2008;16:665-673. 18978289 10.5435/00124635-200811000-00007

[40] Zuckerman JD: Hip fracture. N Engl J Med 1996;334:1519-1525. 8618608 10.1056/NEJM199606063342307

